# Comparative Study of the Dissociative Ionization of 1,1,1-Trichloroethane Using Nanosecond and Femtosecond Laser Pulses

**DOI:** 10.3390/ijms11031114

**Published:** 2010-03-17

**Authors:** Anton du Plessis, Christien Strydom, Lourens Botha

**Affiliations:** 1 CSIR National Laser Centre, PO Box 395, Meiring Naude Road, Pretoria 0001, South Africa; 2 Physics Department, University of Stellenbosch, Private Bag X1, Matieland 7602, South Africa; 3 Chemical Resource Beneficiation, North-West University, Private Bag X6001, Potchefstroom 2520, South Africa

**Keywords:** laser ionization, laser dissociation, coherent control, trichloroethane

## Abstract

Changes in the laser induced molecular dissociation of 1,1,1-trichloroethane (TCE) were studied using a range of intensities and standard laser wavelengths with nanosecond and femtosecond pulse durations. TCE contains C-H, C-C and C-Cl bonds and selective bond breakage of one or more of these bonds is of scientific interest. Using laser ionization time of flight mass spectrometry, it was found that considerable variation of fragment ion peak heights as well as changes in relative peak ratios is possible by varying the laser intensity (by attenuation), wavelength and pulse duration using standard laser sources. The nanosecond laser dissociation seems to occur *via* C-Cl bond breakage, with significant fragmentation and only a few large mass ion peaks observed. In contrast, femtosecond laser dissociative ionization results in many large mass ion peaks. Evidence is found for various competing dissociation and ionization pathways. Variation of the nanosecond laser intensity does not change the fragmentation pattern, while at high femtosecond intensities large changes are observed in relative ion peak sizes. The total ionization yield and fragmentation ratios are presented for a range of wavelengths and intensities, and compared to the changes observed due to a linear chirp variation.

## 1. Introduction

The influence of lasers on molecular processes such as dissociation, ionization and excitation, is aimed at the control of chemical reactions. The laser control of the dissociative ionization of 1,1,1-trichloroethane was investigated using nanosecond and femtosecond laser sources, in order to determine the possibility and extent of control of bond breaking due to nonresonant multiphoton excitation. The resulting trends observed could potentially be applicable to other molecules. Practical application of laser controlled molecular dissociation is more likely when standard commercial laser wavelengths are used.

Femtosecond coherent control has been a topic of increasing interest in the last two decades and numerous experimental studies of the effects of laser control of the dissociation of small molecules in the gas phase have been reported. A good summary of the current status of this research field is given by Dantus and Lozovoy [1], Lozovoy and Dantus [2] and Lozovoy *et al.* [3]. The goal is to find optimal laser properties which can selectively break particular bonds in a molecule. In femtosecond coherent control, temporal and spectral pulse shaping is used to induce changes in the laser-molecule interaction and find optimized solutions for particular bond breaking patterns.

Assion *et al.* [4] studied the dissociative ionization of CpFe(CO)_2_X (X = Cl, Br, I) and found optimized pulse shapes for enhancing and minimizing selective bond breaking of different bonds in these molecules. Damrauer *et al.* [5] found that for the molecule CH_2_BrCl, they could favour the C-Cl bond breakage rather than the C-Br bond breakage by a factor of 1.7/1. Graham *et al.* [6] studied C-C bond breaking in acetophenone (with different C-C bonds which could be broken) with similar results. Cardoza *et al.* [7–9] studied bond breaking in CH_3_COCF_3_, CH_3_COD_3_, CH_3_COCCl_3_ and CH_2_BrI using closed loop coherent control. They found that control over the ratio of CCl_3_/CH_3_ was possible and the optimized and minimized ratios were roughly 1.0 and 0.2 respectively [9].

Numerous studies have been conducted for the purpose of comparing nanosecond and femtosecond laser dissociation and ionization using high intensity laser ionization coupled to time of flight mass spectrometry [10,11]. Weinkauf *et al.* [11] compared 5 ns and 500 fs laser ionization of a series of molecules with the same pulse energies and found identical total ion yields, but different fragmentation patterns. A general trend is that when using femtosecond laser pulses for ionization, the parent molecular ion is observed but not for nanosecond laser ionization.

There is also a fundamental interest in laser-molecule interactions at the high intensities available from femtosecond lasers [12,13]. These papers describe the multiphoton ionization mechanisms termed ladder climbing and ladder switching, which explain the presence of parent molecular ion in ultrashort pulse experiments but more fragmentation in the case of longer pulse durations. Although ladder climbing multiphoton ionization resulting in large parent ion peak formation is true of most experimental high intensity ionization using typical femtosecond lasers in the region of 10^13^–10^15^ W/cm^2^, there are a few exceptions reported in which extensive fragmentation occurs at all intensities and a negligible amount of the parent ion is observed. Harada *et al.* [14] describe this phenomenon with a series of experiments on molecules exhibiting both these extremes and explain this effect as due to resonances with the laser wavelength inducing substantial fragmentation. Other authors dispute this and attribute these differences to physical experimental parameters such as inhomogeneity in the spatial and temporal pulse profile induced by optical materials in the beam path [15]. Although the reasons for the observed differences are not yet known, it is well known in the field that there are considerable differences in reported femtosecond laser mass spectra of identical molecules. At higher intensities (above 10^15^ W/cm^2^), processes such as field ionization resulting in highly charged ions and Coulomb explosions, tunneling ionization and over-the-barrier ionization, may occur. An early review of field ionization and Coulomb explosion of diatomic molecules is given by Posthumus *et al.* [16].

**Figure 1.**
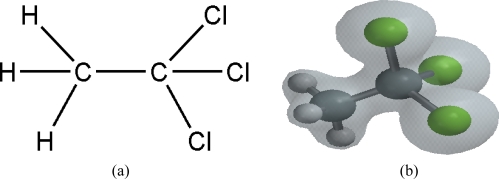
1,1,1-Trichloroethane: **(a)** chemical structure representation and **(b)** electron density distribution.

1,1,1-Trichloroethane (TCE, chemical formula CH_3_CCl_3_) is a chlorinated hydrocarbon comprising a methyl group (CH_3_) bonded to a carbon trichloride group (CCl_3_) through a C-C bond. Its chemical structure and calculated electron density distribution are shown in Figures 1(a) and 1(b). Absorption cross section data reported by Vanlaethem-Meurée *et al.* [17], Nayak *et al.* [18] and Hubrich and Stuhl [19] all show a strong absorption band in the range of 160–190 nm, with a decreasing absorption edge in the deep UV. Spectrophotometer measurements in our laboratory confirmed this absorption band close to 200 nm as well as the lack of any absorption features in the visible or near UV regions. The ionization energy of TCE is reported to be approximately 11 eV, equivalent to 113 nm for single photon ionization [20]. Since this molecule has no visible or near UV absorptions, it is a good example molecule for nonresonant laser dissociative ionization and a study of its control.

The photochemistry, and especially the photo-dissociation pathways of TCE, is not described in the literature, and thermal dissociation pathways are used as a first estimate of the dissociation processes. The pyrolysis and oxidation of TCE was studied by Wu and Bozzelli [21], who found that the primary thermal dissociation pathway when heated to 600 °C is Cl-elimination:

$${\text{CH}}_{3}{\text{CCl}}_{3}\to {\text{CH}}_{3}{\text{CCl}}_{2}\cdot +\text{Cl}\cdot$$

At higher temperatures a second dissociation pathway was observed, due to HCl elimination:

$${\text{CH}}_{3}{\text{CCl}}_{3}\to {\text{CH}}_{2}{\text{CCl}}_{2}+\text{HCl}$$

A third decomposition reaction at high temperatures was identified as:

$${\text{CH}}_{3}{\text{CCl}}_{3}\to {\text{CH}}_{3}\cdot +{\text{CCl}}_{3}\cdot$$

A study of infrared laser photolysis using 10 micron pulsed laser radiation of 1,1,2-trichloroethane showed the main dissociation pathway to be due to HCl elimination [22].

The focus of this present study is on a direct comparison between the nonresonant nanosecond and femtosecond laser dissociation and ionization of TCE using standard wavelengths of the most widely used and available nanosecond and femtosecond pulsed laser sources, namely Nd:YAG nanosecond laser wavelengths of 1,064 nm, 532 nm, 355 nm and 266 nm and Ti:Sapphire femtosecond laser wavelengths of 795 nm and 397 nm. Total ionization yields and fragmentation patterns are reported. The motivation is to investigate the possibility of control over dissociation products using intensity and standard laser pulse durations and wavelengths. The extent of this intensity control should be compared to typical coherent control experiments. In this work the extent of intensity control is compared to an open loop control experiment inducing chirp over a small range, and similar to other workers [3] we find that changes are related to the pulse duration and hence the intensity.

## 2. Experimental Setup

### 2.1. Femtosecond Laser System

The femtosecond laser system is a chirped pulse amplifier (CPA) femtosecond Ti:sapphire laser with central wavelength 795 nm, bandwidth ranging from 6.5 to 10.5 nm FWHM, pulse duration between 130 and 160 fs when transform limited, and with a repetition rate of 1 kHz (Coherent Legend). The CPA is pumped by a 1 kHz frequency doubled Nd:YLF laser (Coherent Evolution) and seeded by a femtosecond Ti:sapphire oscillator (Coherent Mira 900F), which itself is pumped by the *cw* output from a Nd:YVO_4_ laser (Coherent Verdi). The maximum pulse energy from the CPA is 1.05 mJ. The beam has a Gaussian radius w = 3.6 mm at the focusing lens of the time of flight mass spectrometer, and its M^2^ was measured as 1.2 ± 0.15. An uncoated fused silica lens of f = 150 mm was used, which results in focused beam waist radii given in Table 1. The frequency doubled 397 nm radiation was obtained by passing the fundamental 795 nm beam unfocused through a 0.3 mm BBO crystal. The residual 795 nm was filtered out using two filters, one short pass filter with edge at 450 nm and one 50 nm bandpass filter for 400 nm. The resulting maximum pulse energy was approximately 12 μJ. Incorporated in this system is an acousto-optic programmable dispersive filter (FastLite Dazzler) for pulse shaping of the oscillator pulses before amplification. The amplified pulses are characterized using either a home-built second harmonic generation autocorrelator or a Grenouille (SwampOptics).

### 2.2. Nanosecond Laser System

A Continuum Surelite Nd:YAG nanosecond laser with maximum pulse energies at each harmonic: 120 mJ (1064 nm), 32 mJ (532 nm), 6 mJ (355 nm) and 6 mJ (266 nm) was used. The pulse duration was measured as 8 ns and the bandwidth is specified as 1 cm^−1^. Beam radii were estimated from energy transmission through an accurately positioned variable aperture (86% transmission) as 4.0 mm (1,064 nm), 4.0 mm (532 nm), 3.3 mm (355 nm) and 2.5 mm (266 nm).

### 2.3. Time of Flight Mass Spectrometer

A home-built reflectron time of flight mass spectrometer was used. This instrument consists of two chambers separately pumped by two turbomolecular pumps (60 l/s) backed by mechanical rotation pumps. The base pressure of the system in both chambers is approximately 5 × 10^−7^ mbar. Sample gas is introduced effusively through a needle valve and then passed through a skimmer into the ionization region. The unit mass resolution was measured as Δm/m = 240 for peaks of mass 117 amu (CCl_3_^+^), and 232 amu for peaks with mass 36 amu (H^35^Cl^+^). A liquid sample is placed in a stainless steel sample holder connected to the inlet system with Swagelok fittings and frozen with liquid nitrogen while pumping away the residual air above the sample, followed by sample melting. Once this procedure is completed, the needle valve is slowly opened and an equilibrium pressure in the vacuum chamber is eventually reached, determined by the pumping capacity, the vapour pressure of the sample at room temperature and the needle valve setting. The optimum sample pressure, at which space charge effects were not yet observed, but significant signals were obtained, was in the range 2–3 × 10^−5^ mbar in the ionization region. All experiments reported here were kept within this range.

The laser beam was focused using an uncoated lens of f = 150 mm, through the entrance window (UV fused silica 3 mm thick) into the centre of the ionization region. The flight tube axis, molecular beam and laser beam directions are all perpendicular to each other. The lens was mounted in a manual XYZ micrometer stage combination for precise translation of the lens and hence the focus position in the ionization region. Before each experiment, the optimum is found in all these directions manually by observing the mass spectrum.

The laser polarization is linear and parallel to the flight tube and attenuation was done in this work using a set of neutral density filters (double filter wheel). The ionization region is between the TOF repeller plate, which is maintained at 1.4 kV, and the first extraction plate at 960 V. This is followed by a second extraction plate at 0 V. The three plates are separated by 1 cm each and the extraction apertures are 8 mm and 3.5 mm in diameter. This choice was made to ensure all ions generated were collected, irrespective if these were resultant from the highest intensity focus spot, or from regions around the focus at lower intensity (the Rayleigh Range for the 795 beam focused with 150 mm lens was 742 μm). Therefore our mass spectra are integrated over a range of intensities, close to the peak intensity. The reason for this choice was due to our final aim of coherent control of molecular dissociation, not only in the small focus region, but rather achieving control over such a process over the entire focus region. The ions pass through the extraction holes into the field-free flight tube and into the reflectron, which consists of a set of 14 plates with central apertures of 60 mm, except for the final plate without aperture. The voltage difference between the first and final plates is 1.3 kV and the plates in between have intermediate voltages. The reflected ions travel at an angle of 3.5 degrees relative to the original direction until they reach a set of steering plates to which a potential of 500 V is applied, to steer the ions into the detector assembly. This consists of a microchannel plate (Hamamatsu F1552-01) followed by an electron multiplier (Hamamatsu R2362). The signals are collected on a 500 MHz digital oscilloscope (Tektronix) and mass spectra are averaged over 512 for the 1 kHz femtosecond laser and over 64 for the 10 Hz nanosecond laser.

The stability of the entire system is crucial and before each series of experiments, after sufficient warm-up and stabilization, a series of measurements are taken to ensure stability. The standard deviation of the Cl^+^ peak signal in the nanosecond experiment (266 nm, 6 mJ) was 5.7% for 10 measurements over 1 hour. In a similar femtosecond experiment over 1 hour (795 nm, 1 mJ) this value was 3.4%.

As a check of the calculated laser intensity, the ionization of Helium was studied as a function of laser intensity with the 795 nm femtosecond laser over the intensity range 1 × 10^14^ to 3 × 10^15^ W/cm^2^. The observed ionization threshold is approximately 1 × 10^15^ W/cm^2^, which is in good agreement with that of Lozovoy *et al.* [3] who found a value of 1.29 × 10^15^ W/cm^2^.

The focal spot size for 795 nm ionization is 13.7 μm, with a corresponding Rayleigh range of 742 μm. The ionization volume above 1 × 10^14^ W/cm^2^ was calculated using the formula in Posthumus [23] as 6.8 × 10^−7^ cm^3^. At our pressures this results in an estimated 4 × 10^5^ ions, similar to that reported by Lozovoy *et al.* [3]. Space-charge effects could be observed at higher pressures in the form of peak broadening on all peaks, but this situation was avoided in these experiments.

## 3. Results

### 3.1. Nanosecond Ionization

Nanosecond laser ionization was attempted with all four fundamental Nd:YAG laser wavelengths at maximum pulse energies. No ionization was achieved using the available pulse energy of the 1064 nm wavelength, at a peak intensity of 2.6 × 10^12^ W/cm^2^ and fluence 2.09 × 10^4^ J/cm^2^.

#### 3.1.1. Nanosecond Ionization 532 nm

**Figure 2.**
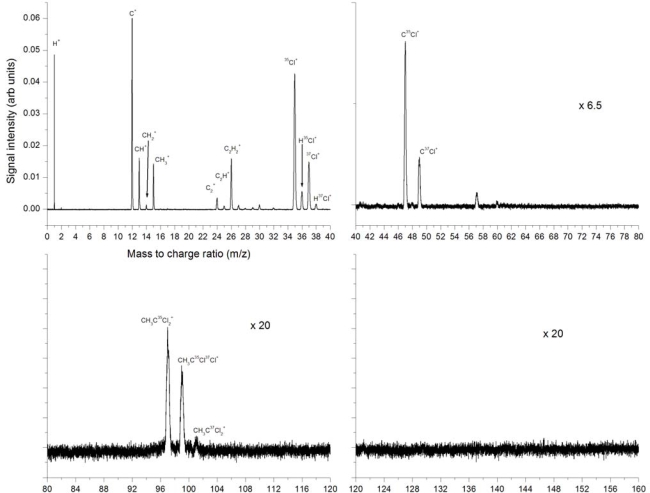
Mass spectra from laser ionization and dissociation of trichloroethane using 532 nm, 8 ns pulses at a peak intensity of 2.8 × 10^12^ W/cm^2^ (Fluence 2.25 × 10^4^ J/cm^2^ per pulse).

A detailed presentation of the 532 nm ionization at 2.8 × 10^12^ W/cm^2^ is given in Figure 2 for separate mass sections and includes identification of the most important peaks. The strongest peak is that of C^+^, second is H^+^ and third is ^35^Cl^+^. The peaks CH^+^, CH_2_^+^ and CH_3_^+^ are identified. The CH_3_^+^ peak could be due to bond breaking of the C-C bond in neutral or ionized CH_3_CCl_3_ but could also result *via* other dissociation and dissociative ionization pathways, for example sequential dissociation of chlorine atoms, the C atom and subsequent ionization. The H^+^ peak is clearly the result of many possible dissociation and ionization pathways, as is the C^+^ peak. Also visible here are the C_2_^+^, C_2_H^+^, C_2_H_2_^+^ and C_2_H_3_^+^ peaks. The C_2_H_3_^+^ peak could be the result of the dissociation of the 3 chlorine atoms from neutral or ionized CH_3_CCl_3_. The ^35^Cl^+^ and ^37^Cl^+^ isotopes are clearly distinguishable, as are the HCl^+^ combinations. These indicate HCl elimination as a possible dissociation pathway. In the higher mass region we observe CCl^+^ peaks of the two isotopes of chlorine. CCl^+^ could be the result of a number of potential dissociation pathways. At masses 97, 99 and 101, the chlorine isotopes of CH_3_CCl_2_^+^ are identified, although these are weak. These are the result of the dissociation of a single chlorine atom from the neutral or ionized parent molecule. No peaks are observed in the higher mass region where the parent molecular peak would be expected at mass 132 for the most abundant chlorine isotope.

**Figure 3.**
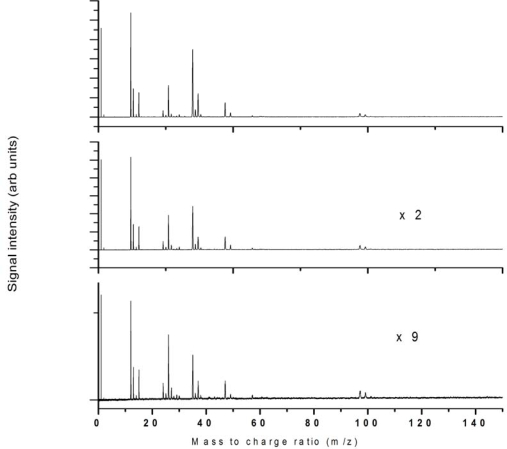
532 nm ionization at laser intensities **(a)** 2.8 × 10^12^ W/cm^2^, **(b)** 1.8 × 10^12^ W/cm^2^ and (c) 1.1 × 10^12^ W/cm^2^

The intensity dependence of 532 nm ionization was studied up to 2.8 × 10^12^ W/cm^2^ and the fragmentation pattern was reasonably unchanged across this intensity regime as shown in Figure 3 for 3 intensities across this range as indicated.

**Figure 4.**
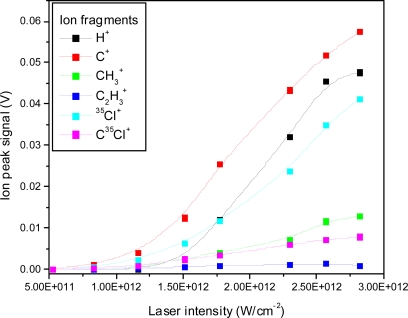
Ion peak signals as a function of laser intensity for H^+^, C^+^, CH_3_^+^, C_2_H_3_^+^, ^35^Cl^+^, C^35^Cl^+^ fragment ions using 532 nm laser radiation with 8 ns pulse duration.

The ion peak signals as a function of laser intensity for the C^+^, CH_3_^+^, C_2_H_3_^+^, ^35^Cl^+^ and C^35^Cl^+^ ion peaks are shown in Figure 4. The fitted lines are for visual appearance. It is interesting to note relative changes, such as the relative faster increase of the H^+^ ion peak in comparison to the slower increase of the ^35^Cl^+^ ion peak. Furthermore, the slopes are different for different fragments, also indicating changes in the relative fragmentation ratios.

#### 3.1.2. Nanosecond Ionization 355 nm

**Figure 5.**
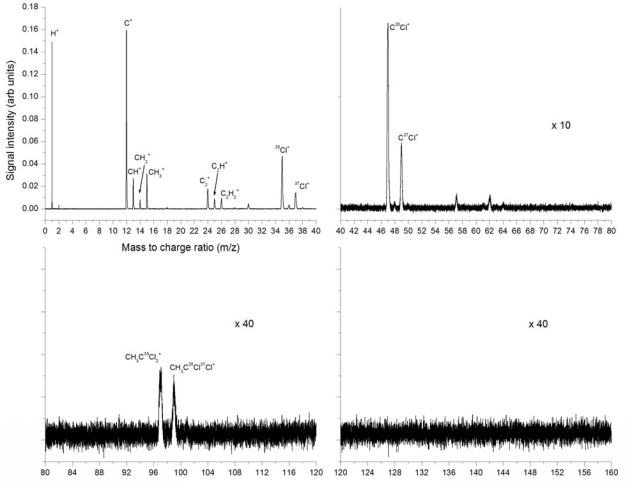
Mass spectra from laser ionization and dissociation of trichloroethane using 355 nm, 8 ns pulses at 6.8 × 10^11^ W/cm^2^ (Fluence 5.4 × 10^3^ J/cm^2^ per pulse).

Figure 5 shows the 355 nm ionization at 6.8 × 10^11^ W/cm^2^. It is clear that all peaks have roughly the same ratio as in the 532 nm case. The strongest peaks are H^+^ and C^+^, followed by the Cl^+^ isotopes. H_2_^+^ is observed which was not observed at 532 nm, the CCl^+^ peaks are clear as well as the peak at mass 62 which can be explained as the CH_3_CCl^+^ fragment, although it is very weak. The CH_3_CCl_2_^+^ peaks are observed as before, but no higher mass peaks are observed. The observation of CH_3_CCl_2_^+^ and CH_3_CCl^+^ peaks, and lack of other high mass peaks, identifies sequential Cl dissociation as a possible first dissociation reaction, before further fragmentation occurs. The lack of parent ion peak also indicates that this Cl-dissociation could take place before ionization, resulting in neutral dissociation followed by ionization of the fragments.

**Figure 6.**
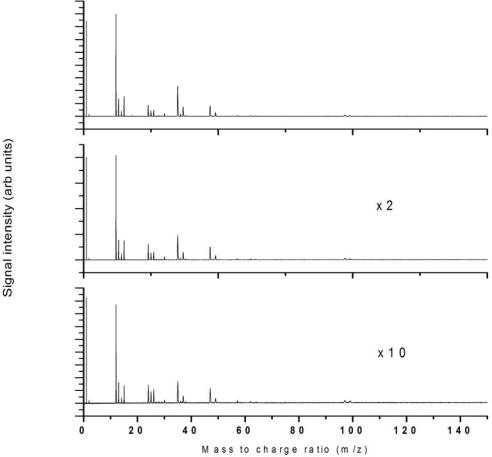
355 nm ionization using laser pulse energies of 6.8 × 10^11^ W/cm^2^, 4.6 × 10^11^ W/cm^2^ and 2.3 × 10^11^ W/cm^2^.

**Figure 7.**
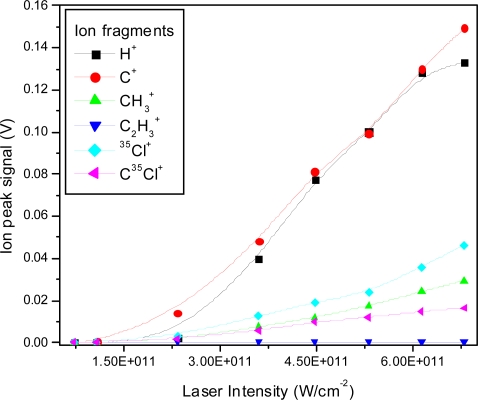
Ion peak signals as a function of laser intensity for H^+^, C^+^, CH_3_^+^, C_2_H_3_^+^, ^35^Cl^+^, C^35^Cl^+^ fragment ions using 355 nm laser radiation with 8 ns pulse duration.

The intensity dependence of 355 nm ionization was studied up to 6.8 × 10^11^ W/cm^2^ and the fragmentation pattern was reasonably unchanged across this intensity regime as shown in Figure 6 for three intensities across this range as indicated. Ion peak signals as a function of laser intensity are presented in Figure 7.

#### 3.1.3. Nanosecond Ionization 266 nm

**Figure 8.**
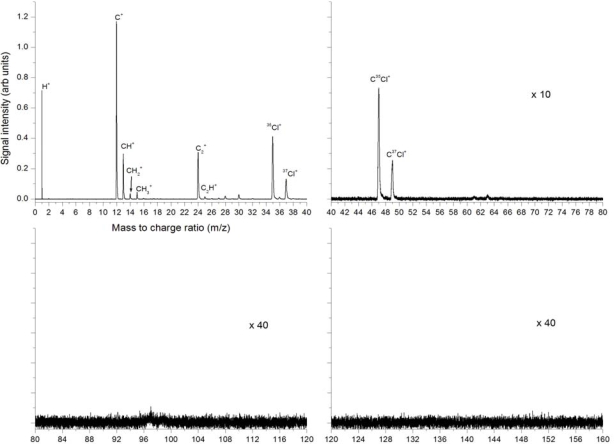
Mass spectra from laser ionization and dissociation of trichloroethane using 266 nm, 8 ns pulses at 9 × 10^11^ W/cm^2^ (Fluence 7.2 × 10^3^ J/cm^2^).

Figure 8 shows the 266 nm ionization at 9 × 10^11^ W/cm^2^. The fragmentation pattern is similar to that seen in Figures 3 and 5, with mostly low mass peaks present and very few and low intensity higher mass peaks. The strongest peaks are H^+^, C^+^, ^35^Cl^+^ and ^37^Cl^+^. All peaks present in the other nanosecond experiments presented above are also present here in roughly the same ratios. In particular the C^+^ > CH^+^ > CH_2_^+^ but the CH_3_^+^ peak is in this case only slightly larger than CH_2_^+^, not as much larger than in the other nanosecond experiments. C_2_^+^ is clearly visible as before but C_2_H^+^, C_2_H_2_^+^ and C_2_H_3_^+^ are much weaker in relation. Also the HCl^+^ peaks are much weaker than in the other experiments. The CCl^+^ peaks are strong but there are no higher mass peaks observed. In this case, in contrast to the experiments at 355 nm and 532 nm, we do not observe the large mass peaks of CH_3_CCl_2_^+^ at 99, 101 and 103 amu. These results indicate that stronger fragmentation has occurred under these conditions, in comparison to 355 nm and 532 nm.

**Figure 9.**
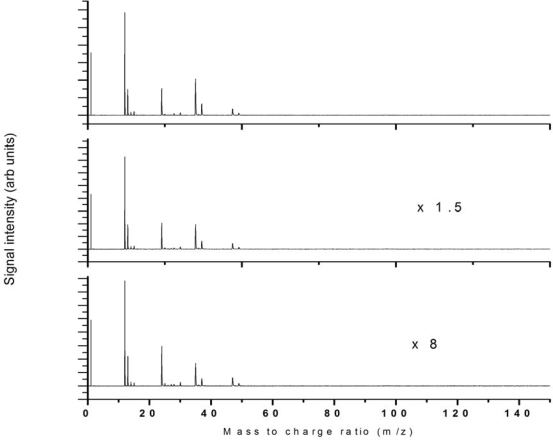
355 nm ionization using laser pulse energies of 9 × 10^11^ W/cm^2^, 5.5 × 10^11^ W/cm^2^ and 2.1 × 10^11^ W/cm^2^.

**Figure 10.**
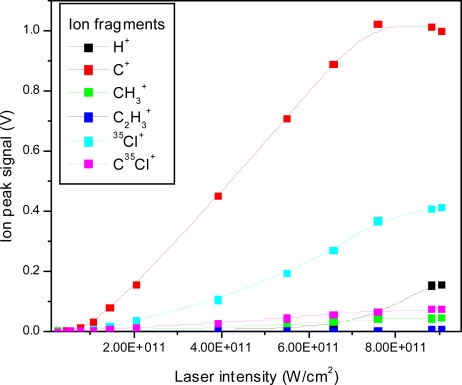
Ion peak signals as a function of laser intensity for H^+^, C^+^, CH_3_^+^, C_2_H_3_^+^, ^35^Cl^+^, C^35^Cl^+^ fragment ions using 266 nm laser radiation with 8 ns pulse duration.

The intensity dependence of 266 nm ionization was studied up to 9 × 10^11^ W/cm^2^ and the fragmentation pattern was unchanged across this intensity regime as shown in Figure 9 for 3 intensities across this range as indicated. Ion peak signals as a function of laser intensity are presented in Figure 10.

### 3.2. Femtosecond Ionization

#### 3.2.1. Femtosecond 795 nm Ionization

**Figure 11.**
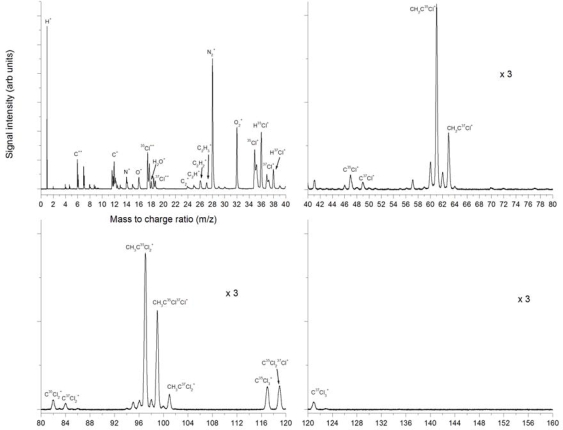
Mass spectra from laser ionization and dissociation of trichloroethane using 795 nm, 150 fs pulses at 2.3 × 10^15^ W/cm^2^ (Fluence 350 J/cm^2^).

Femtosecond ionization at the fundamental 795 nm wavelength up to 2.3 × 10^15^ W/cm^2^ resulted in mass spectra as shown in Figure 11. Qualitatively it is clear that the fragmentation is entirely different than in the nanosecond experiments and that more higher mass fragments are visible, as well as a larger number of smaller mass fragments. A strong H^+^ peak is observed, as well as C^+^, C^2+^ and C^3+^. Background gas peaks are considerably intense with peaks of N^+^, O^+^ and H_2_O^+^ identified, as well as very strong molecular N_2_^+^ and O_2_^+^ peaks. Also identified are the ^35^Cl^+^ and ^37^Cl^+^ peaks and the corresponding HCl^+^ peaks, as well as C_2_^+^, C_2_H^+^, C_2_H_2_^+^ and C_2_H_3_^+^. It is interesting to note that highly charged ions are observed for some species.

As mentioned above, doubly and triply charged C is observed. Also observed are doubly charged Cl ions of both isotopes around the mass 18. Triply charged Cl is also observed around mass 12 as well as Cl^4+^ around m/z = 9. Of particular interest is the peak splitting observed on some peaks, in particular on the Cl^+^ peaks (but not the HCl^+^ peaks), H^+^, N^+^, O^+^ and on the highly charged ion peaks described above. Peak splitting is indicative of explosive dissociation, and can be due to a Coulomb explosion process occurring at high intensities. In this case the Coulomb explosion of the parent molecule or large fragments can result in these small mass peaks such as Cl^+^. The splitting occurs due to the ions which have initial velocity components in the direction of the flight tube and those in the opposite direction which are also collected. However, no peak splitting is observed on any of the large mass molecular fragments. Therefore the first dissociation processes resulting in fragment ions as observed do not seem to occur explosively. Furthermore, the lack of a parent molecular peak is interesting and indicates the possibility that the first dissociation process takes place from the neutral parent molecule. The higher mass peaks of CCl^+^ are observed similar to the previous nanosecond experiments, but also observed are very strong peaks of CH_2_CCl^+^ and combinations of these with varying numbers of H’s attached. It is interesting to note that the CH_2_CCl^+^ peak is stronger than the CH_3_CCl^+^ peak. This is an indication that a direct dissociation process resulting in this particular fragment could be dominant. This can possibly be explained by direct HCl elimination from CH_3_CCl_2_. In this figure we observe CCl_2_^+^ peaks, probably arising from dissociation of the CCl_3_ fragments (starting at the higher mass of 117 amu). The CH_3_CCl_2_^+^ peaks are also observed very strongly with different combinations of H’s attached. The CH_3_CCl_2_^+^ is a direct dissociation fragment by the dissociation of a single chlorine atom from the parent molecule or ion. No parent molecular peak is observed at the expected mass 132.

**Figure 12.**
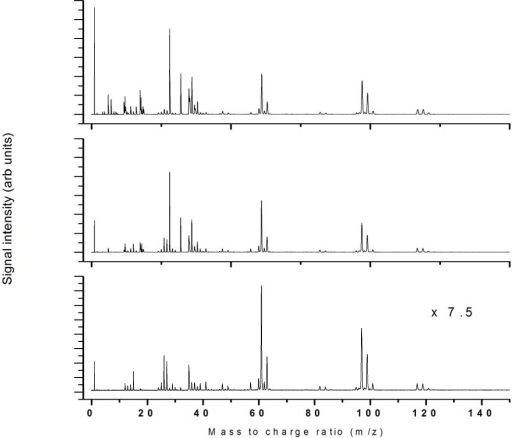
795 nm ionization using 2.3 × 10^15^ W/cm^2^, 5.6 × 10^14^ W/cm^2^ and 1.3 × 10^14^ W/cm^2^.

Mass spectra recorded as a function of laser intensity show significant changes in relative fragment peaks, although all major peaks identified above are present at lower intensities and no additional peaks are observed. Mass spectra at three different intensities are shown in Figure 12.

**Figure 13.**
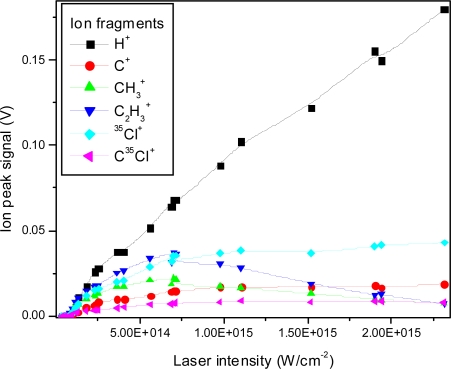
Ion peak signals as a function of laser intensity for H^+^, C^+^, CH_3_^+^, C_2_H_3_^+^, ^35^Cl^+^, C^35^Cl^+^ fragment ions using 795 nm laser radiation with 150 fs pulse duration.

Although the absolute signals of all peaks seem to decrease with decreasing intensity as expected, some relative changes are observed qualitatively. For example in the lowest intensity spectrum shown here, the mass 61 ion (CH_2_CCl^+^) becomes dominant compared to all other peaks, which is not the case at higher intensities. The change in peak sizes is shown quantitatively in Figure 13 for the same low mass ions studied in the nanosecond case.

**Figure 14.**
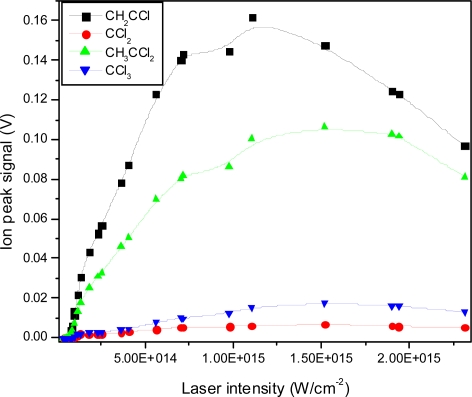
Ion peak signals as a function of laser intensity for CH_2_C^35^Cl^+^, C^35^Cl_2_^+^, CH_3_C^35^Cl_2_^+^, C^35^Cl_3_^+^ fragment ions using 795 nm laser radiation with 150 fs pulse duration.

In addition, the intensity dependence of the heavy mass ion signals are plotted in Figure 14. The threshold for ionization is observed just above 1 × 10^14^ W/cm^2^ followed by an increase and finally, above 1 × 10^15^ W/cm^2^ a decrease is observed for the heaviest mass fragments. This decrease may be explained by an increase in dissociation of these fragments, which would result in a relatively sharper increase in low mass peaks. However, the onset of Coulomb explosion of small mass fragments and the resulting splitting of these peaks results in difficulty in quantifying the increase in these small mass peak signals. In addition, the explosive dissociation process results in a loss of ions due to velocity components in directions other than the flight tube, also resulting in a lower measured yield of small mass peaks.

#### 3.2.2. Femtosecond 397 nm Ionization

**Figure 15.**
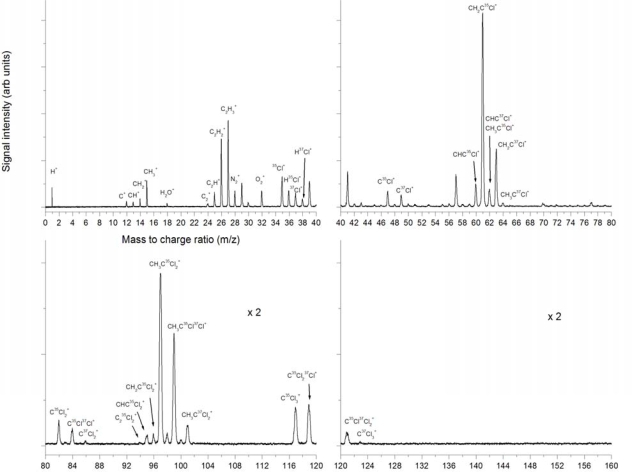
Mass spectra from laser ionization and dissociation of trichloroethane using 397 nm, approximately 150 fs pulses at 7.6 × 10^13^ W/cm^2^ (Fluence 1 J/cm^2^).

The observed mass spectrum when using 397 nm pulses of approximately 150 fs duration at intensity 7.6 × 10^13^ W/cm^2^ is shown in Figure 15. The fragmentation pattern seems very similar to the 795 nm case although the total ionization yield is an order of magnitude lower, mainly due to the laser intensity, which is two orders of magnitude lower. All the peaks present in the 397 nm spectrum are also present in the 795 nm case. No peak splitting is observed as in the 795 nm case. The most important qualitative result is the dominance of the peak at mass 61, which also dominates in low intensity 795 nm ionization. This indicates the possibility that the dissociation processes are very similar in the 795 nm and 397 nm experiments. As before, no parent molecular peak is observed.

**Figure 16.**
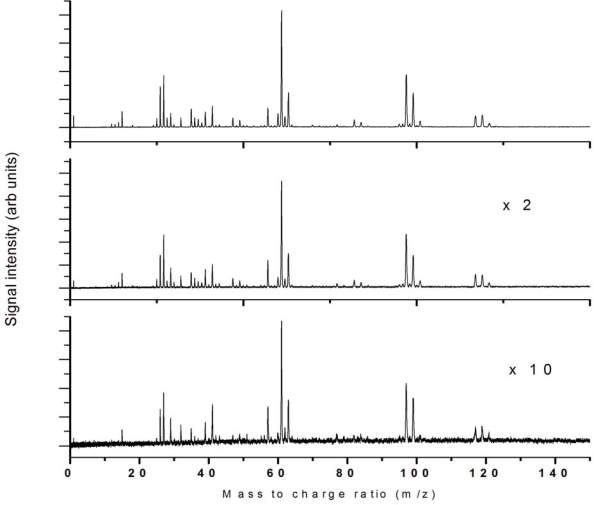
397 nm ionization using 7.6 × 10^13^ W/cm^2^, 4.9 × 10^13^ W/cm^2^ and 3.1 × 10^13^ W/cm^2^.

The mass spectra recorded at three different intensities in the available range (a relatively small range) show almost identical mass spectra, as shown in Figure 16.

**Figure 17.**
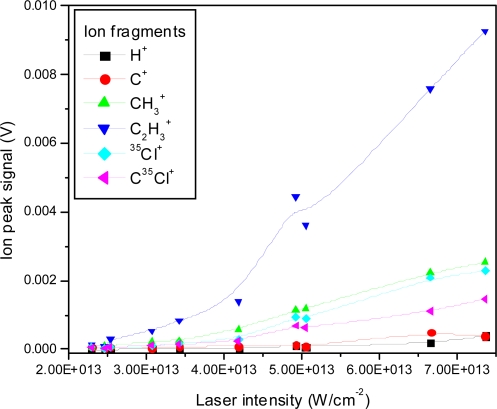
Ion peak signals as a function of laser intensity for H^+^, C^+^, CH_3_^+^, C_2_H_3_^+^, ^35^Cl^+^, C^35^Cl^+^ fragment ions using 397 nm laser radiation with 150 fs pulse duration.

**Figure 18.**
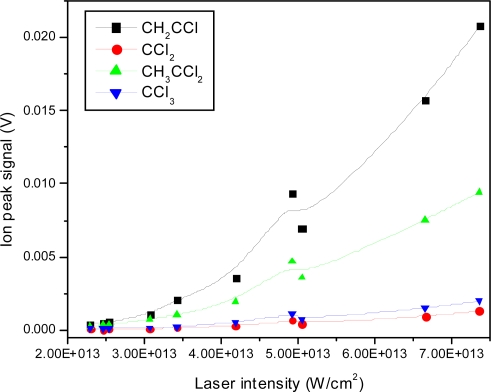
Ion peak signals as a function of laser intensity for CH_2_C^35^Cl^+^, C^35^Cl_2_^+^, CH_3_C^35^Cl_2_^+^, C^35^Cl_3_^+^ fragment ions using 397 nm laser radiation with 150 fs pulse duration.

The intensity dependence of small mass ion peaks are shown in Figure 17, and the same intensity dependence is presented for heavy mass ions in Figure 18. The threshold for ionization is observed at approximately 2.5 × 10^13^ W/cm^2^ followed by a reasonably linear increase. As observed before, the slopes differ, resulting in different fragmentation ratios as a function of laser intensity.

#### 3.2.3. Explosive Dissociation

**Figure 19.**
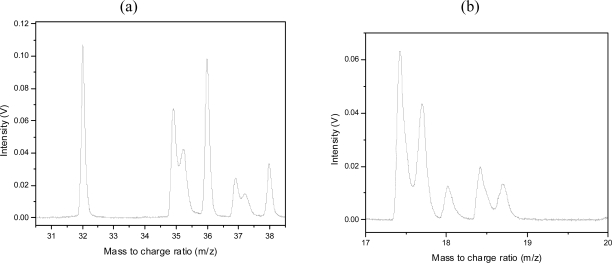
**(a)** Peak splitting of the ^35^Cl^+^ and ^37^Cl^+^ peaks but not the HCl^+^ peaks or the O_2_^+^ peak. **(b)** Peak splitting around mass 18. These are the ^35^Cl^2+^, H_2_O^+^ and ^37^Cl^2+^ peaks.

Peak splitting was observed on the ion peaks H^+^, C^+^, N^+^, O^+^ and Cl^+^ and all higher charged species. A closer look at the observed splitting in our experiment is provided in Figures 19 (a) and (b). The reason for the difference in the intensities of the two peaks of a “split peak” is due to the geometry of ion collection, which is more efficient for ions explosively emitted with a velocity component in the direction of the flight tube. In contrast the ions emitted in the opposite direction must first be stopped and then repelled in the opposite direction into the flight tube by the repeller plate, in this process more ions are lost. This effect is clearly distinguished from space-charge effects, which was observed at higher gas pressures and resulted in broadening of peak (not split peaks) and this broadening was observed on all peaks (not as here with selective split peaks while others remain unaffected). The doubly ionized Cl peaks are shown in Figure 19 (b).

**Figure 20.**
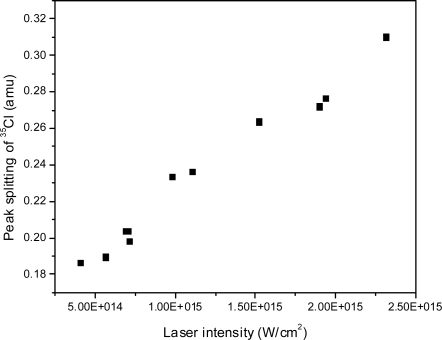
Peak splitting as a function of laser intensity as shown here for the Cl peak. Note the peak width of an “unsplit peak” is 0.16 amu close to this mass, below which no splitting can be observed.

The peak splitting was studied for the ^35^Cl^+^ ion peak as a function of laser intensity as shown in Figure 20: as the intensity increases, the peak-splitting increases linearly. Below approximately 4.5 × 10^14^ W/cm^2^, no peak splitting is observed within of the resolution of our instrument. The increase in split peak amount with increasing intensity may be explained by an increase in the number of ions generated in the ionization volume. As the number of ions increases, each ion is subjected to a larger Coulomb force, resulting in a larger velocity in directions away from the ionization region. As the velocity increases, the amount of splitting increases. The presence of multiply charged ions as observed indicates the presence of multiply charged explosive precursor ions. This could also result in increased peak splitting, in which case higher charge precursors will result in larger Coulomb forces. In our experiment, we find the thresholds for the observation of various ^35^Cl charge states to be 7.8 × 10^13^ W/cm^2^ (^35^Cl^+1^), 1.8 × 10^14^ W/cm^2^ (^35^Cl^+2^), 2.3 × 10^14^ W/cm^2^ (^35^Cl^+3^), 7 × 10^14^ W/cm^2^ (^35^Cl^+4^). No higher charge states for ^35^Cl or ^37^Cl were observed.

#### 3.2.4. Open loop Control Example with Linear Chirp

**Figure 21.**
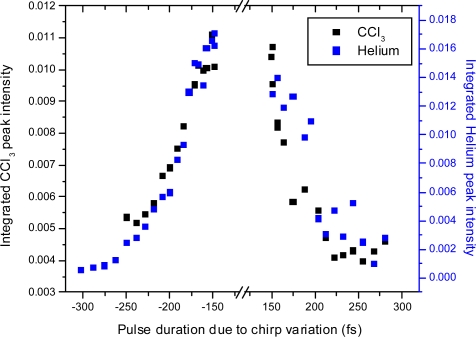
Integrated CCl_3_^+^ and He^+^ peak signal as a function of linear chirp variation for two experiments. Pulse durations due to negative chirp are indicated with negative values, in order to demonstrate the expected symmetry around the shortest pulse duration.

**Figure 22.**
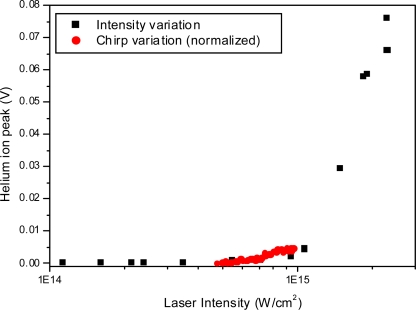
He^+^ peak signal as a function of intensity for two experiments – variation of the intensity using neutral density filters and variation of the chirp resulting in intensity variation (pulse energy kept constant). The chirp variation experiment is normalized using the shortest pulse value (at zero chirp).

Linear chirp was introduced using a Dazzler acousto-optic programmable dispersive filter and the resulting mass spectra recorded. Two experiments were carried out, one with pure helium and one with trichloroethane. The Helium experiment may be seen as a reference experiment, and the only changes expected in ion signal of He^+^ are due to the decreasing laser intensity with increasing linear chirp. In both cases the chirp was adjusted over a range of chirp settings both positive and negative corresponding to pulse durations from 150 fs up to approximately 300 fs. The total pulse energy was kept constant (400 μJ per pulse, equivalent to 9.2 × 10^14^ W/cm^2^ at 150 fs) using neutral density filters (to within 5–10%). The results over the range studied here (for both He^+^ in the Helium experiment and for the CCl_3_^+^ peak in the trichloroethane experiment) are shown in Figure 21 - note the negative pulse durations refer to negative chirp for visual appearance. This figure shows changes mainly related to the effective change in intensity induced by the chirp, as indicated by the symmetry. The integrated He^+^ peak intensity is maximum at the shortest pulse setting and decreases towards the higher chirp settings. The CCl_3_^+^ peak shows the same behaviour and varies by a factor of 3 in this range. Other peaks in the trichloroethane mass spectra vary similarly but by smaller margins. In Figure 22 this variation of intensity due to chirp variation is directly compared to intensity variation using neutral density filters for Helium ionization. Although the variation in intensity due to chirp only covers a small range, the results agree well with one another.

The CCl_3_^+^ / CH_3_^+^ ratio in the chirp experiment was calculated in the same manner as for the intensity variation study. This is an example of a ratio for controlled dissociation, similar to Cardoza *et al.* [9]. This ratio varies from a maximum value at the transform limited case of 1.5 to a minimum value at the strongest chirp of 0.8, for both positive and negative chirp. The reason for these changes are that the CH_3_^+^ value changes much less than the CCl_3_^+^ value over this range. This demonstrates a reasonable amount of control induced by the chirp variation, although no unexpected ratio changes are observed in this range.

## 4. Discussion

**Figure 23.**
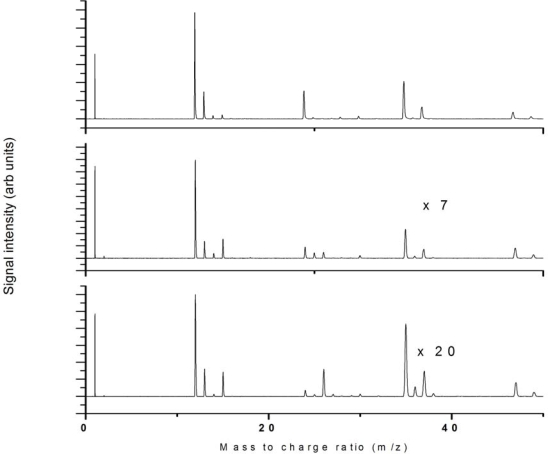
Comparison of nonresonant nanosecond laser ionization using: **(a)** 266 nm, 9 × 10^11^ W/cm^2^, **(b)** 355 nm, 6.8 × 10^11^ W/cm^2^, **(c)** 532 nm, 2.8 × 10^12^ W/cm^2^.

A comparison of the nanosecond ionization mass spectra for the three wavelengths 532 nm, 355 nm and 266 nm is presented in Figure 23 for direct comparison. Qualitatively it is clear that the dissociation and ionization processes are very similar in these experiments, with a few minor exceptions. One trend observed is that longer wavelengths provide small amounts of higher mass peaks (the CH_3_CCl_2_^+^ peaks), in contrast to the 266 nm case in which case these peaks are not present. The observed spectra are similar to those reported by Ma *et al.* [24] for chloro-ethylenes, with almost identical fragmentation patterns and the CCl^+^ peaks as the heaviest mass ions detected. Since the Cl^+^ peaks are strong in all these cases, as well as CH_3_^+^ and C_2_H_3_^+^, the dominant primary dissociation reaction could be C-Cl bond breaking, followed by further dissociation and ionization pathways to produce the observed peaks. The small amounts of the heavy mass CH_3_CCl_2_^+^ observed, and the lack of any other heavy mass fragments, confirm this proposed mechanism, as does the lack of parent ion peak.

**Figure 24.**
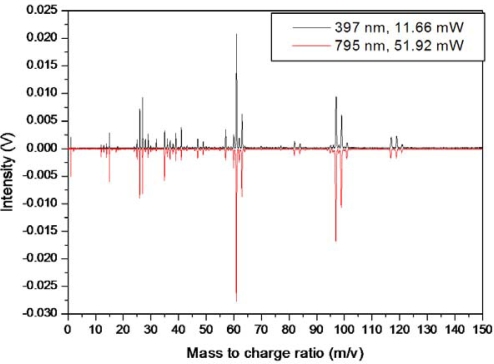
Comparison of femtosecond dissociation and ionization of trichloroethane at low intensities (1.2 × 10^14^ W/cm^2^ for 795 nm and 7.3 × 10^13^ W/cm^2^ for 397 nm). Note the 795 nm mass spectrum is plotted negatively for visual comparison.

As shown in Figure 24, the femtosecond ionization fragmentation pattern seems identical for low intensity 795 nm and 397 nm. This direct comparison is made by a choice of laser intensities such that the Cl^+^ peaks are similar. This confirms the identical fragmentation pattern and indicates a striking similarity in the dissociative ionization for the two wavelengths. It is due to this striking similarity that particular care was taken to ensure only 397 nm component in the beam, using a combination of 2 filters.

In a study of the femtosecond dissociative ionization of halogenated ethylenes by Castillejo *et al.* [25], extensive fragmentation at both 800 and 400 nm was observed and peak splitting indicating Coulomb explosion, of the Cl and C cations with charges up to 4+ and 3+ respectively was observed. This is identical to the results reported here (at higher intensities using 795 nm), even up to the same charge states for the two species. Similar Coulomb explosions were observed for a range of aromatic molecules and their deuterated derivatives by Tzallas *et al.* [26]. Recent work on the Coulomb explosion of alkyl halide molecules (C_2_H_5_Cl, C_3_H_7_Cl and C_4_H_9_Cl) by Kaziannis and Kosmidis [27] show the explosion process to have a strong angular distribution relative to the laser polarization direction. Another interesting study indicates the possibility that explosive dissociation does not necessarily occur as a Coulomb explosion but neutrals may also explosively dissociate [28].

**Figure 25.**
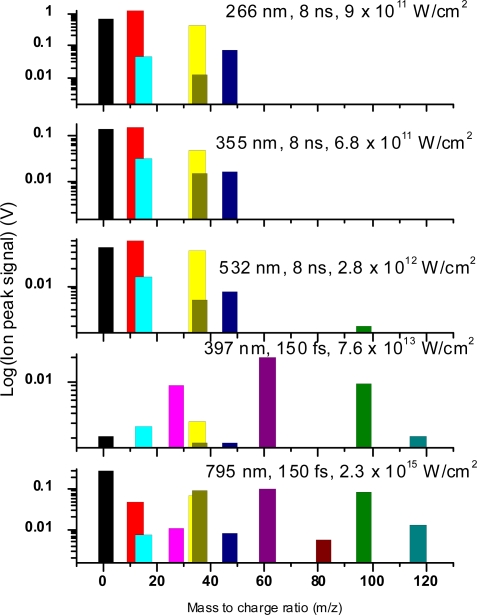
Absolute signals of the most important ion peaks for the various laser wavelengths, from top to bottom: 266 nm, 355 nm, 532 nm (nanosecond) and 397 nm and 795 nm (femtosecond). These values are from the highest laser intensity in each case.

Figure 25 shows the ion signals of the most important ion fragments for each wavelength studied, at the highest laser intensity in each case. This shows the differences in ion signals for the different experiments. One of the major differences is the lack of large mass fragments in the nanosecond spectra. Furthermore, relative changes in various ion signals are observed clearly.

**Figure 26.**
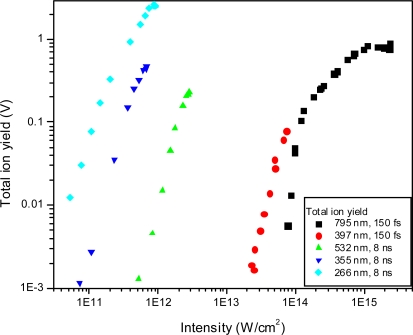
Total ion yield (summation over relevant dissociation product ions) as a function of laser intensity for all laser wavelengths and pulse durations employed.

From this data, the total ionization can be estimated in each experiment by summation over these most important ion peaks. This total ionization yield as a function of laser intensity for all the wavelengths studied is shown in Figure 26. This confirms quantitatively the observed trend that the highest ionization efficiency is produced by the nanosecond 266 nm ionization with a total ionization value of 2.8, second is the femtosecond 795 nm with 0.7, followed by nanosecond 355 nm with 0.5 and 532 nm with 0.2, and finally femtosecond 397 nm with 0.06. These are calculated at the highest available pulse energies. In the case of multiphoton ionization, these ion yields as a function of laser intensity are expected to provide straight lines (on a log-log plot) with slopes related to the order of the nonlinear process. However, since the ionization energy is 11 eV, we would expect a minimum of 3 photons to be required for 266 nm, 4 photons for 355 nm and 6 photons for 532 nm. However the slopes for the yield as a function of laser intensity for these three wavelengths are very similar and roughly equal to 3. This result demonstrates the complexity of the dissociative ionization processes, and the lack of information of the many possibly competing pathways. In the femtosecond experiments at 795 nm, 7 photons would be required while at 397 nm at least 4 photons would be required in multiphoton ionization. Straight lines could not be fit to the femtosecond data, indicating the expected result that multiphoton ionization is not the ionization mechanism, but rather strong-field ionization.

Cardoza *et al.* [9] reported closed loop coherent control of the CCl_3_^+^/CH_3_^+^ ratio from dissociative ionization of trichloroacetone (CH_3_COCCl_3_) with optimized and minimized ratios of roughly 1.0 and 0.2 respectively. In addition they note that no parent molecular peak is observed for this molecule, although a strong parent molecular peak is observed for trideuterated acetone. In our experiment, even though the molecule is different, this same ratio was chosen as an example to indicate the relative changes that can be induced in the fragmentation process. We found that this ratio (CH_3_^+^/CCl_3_^+^) is strongly dependent on laser intensity and varies from 0.35 to 1.72 over the range of 795 nm intensities studied here, and from 0.74 to 0.92 over the range of 397 nm intensities. The same ratios for our nanosecond experiments are zero due to the lack of CCl_3_^+^ peaks. Similarly the chirp variation experiment produced changes in this ratio of 0.8 to 1.5.

## 5. Conclusions

The trichloroethane parent molecular peak was not observed in any of the experiments reported here. The lack of parent ion indicates the possibility that a primary dissociation reaction takes place from the neutral molecule. In the nanosecond regime, indications are that Cl dissociation is the primary reaction due to the presence of CH_3_CCl_2_^+^ in some of these spectra and lack of any other large fragments, and also due to the strong Cl^+^ peaks observed. In the femtosecond experiments at low intensity there are numerous large fragments, most notably CCl_3_^+^, CH_3_CCl_2_^+^ and CH_2_CCl^+^. The dominance of the CH_2_CCl^+^ peak identifies direct HCl elimination from CH_3_CCl_2_ as a possible dissociation reaction after Cl dissociation from the parent molecule. The presence of CCl_3_^+^ does indicate that some C-C bond breaking may occur as a primary dissociation reaction. These speculations are complicated by the various possibly competing ionization and dissociation pathways, including dissociation from the neutral parent molecule, dissociation of these fragments (sequentially), ionization of the parent molecule followed by dissociation from the ionized parent molecule, as well as different mechanisms of excitation and ionization. Ionization mechanisms include multiphoton ionization (most likely non-resonant due to the lack of absorption features at the wavelengths employed here), as well as strong-field ionization mechanisms such as field ionization.

Extensive fragmentation is observed in all experiments reported here (both nanosecond and femtosecond), and this fragmentation varies considerably as a function of laser intensity. At the highest intensities in the femtosecond experiment, highly charged atomic ions and peak splitting was observed on numerous peaks. This is explained by Coulomb explosion of larger fragments. Comparison between nanosecond and femtosecond dissociative ionization at lower intensities show an increased amount of fragmentation at nanosecond pulse durations.

In terms of laser control of dissociation, an example ratio of CCl_3_^+^/CH_3_^+^ taken from these measurements yield strong variation with laser intensity, and some degree of variation but less than the intensity over a small range of linear chirp values induced in the 795 nm femtosecond laser experiment. As another example of control, the absolute signal size (related to the total number of dissociated fragment ions) of various ion peaks (such as C^+^ and Cl^+^) are strongest in the 266 nm experiment, even though the intensity is 3 orders of magnitude lower than the 795 nm femtosecond experiment.

In conclusion, trichloroethane dissociation and ionization efficiency is higher with nanosecond laser pulses of 8 ns and most efficient at shortest wavelengths (266 nm compared to 355 nm, 532 nm and 1064 nm). In the nanosecond experiment, the increased efficiency of ionization of 266 nm is expected due to the lower number of photons required, and the lower order of the expected multiphoton ionization mechanism. However, even though the thresholds are higher, the same order of nonlinear process is found for the longer wavelengths – this may be explained by relatively increased efficiency above that expected due to resonances with dissociative states or states in the neutral or ionized fragments. In the femtosecond experiment, the intensity regime covers values of the Keldysh parameter ranging from roughly γ = 1 (between multiphoton ionization and tunneling ionization, more likely the latter) to γ = 0.2 (solidly in the strong-field regime of over-the-barrier ionization). This results in entirely different fragmentation patterns. In particular, the difference is the lower amount of fragmentation, which may be useful if selective bond breaking is the goal, for example C-C bond breakage may yield CH_3_ and CCl_3_ fragments followed by ionization. The fragmentation pattern shows dependences on laser intensity and chirp. Such changes can be manipulated to either optimize a relevant peak ratio, or optimize one or more fragment ion peaks. This is demonstrated for the CCl_3_^+^/CH_3_^+^ ratio over a wide range of intensities (ratio 0.35 to 1.72) and over a linear chirp variation (ratio 0.8 to 1.5). The implications for laser control of molecular dissociation is that both nanosecond and femtosecond lasers provide a reasonable variation of the mass spectra (which may be seen as a form of control over the dissociative ionization processes) using only intensity and wavelength.

## Figures and Tables

**Table 1.** Laser parameters.

**Pulse duration**	**Wavelength (nm)**	**Max pulse energy (mJ)**	**Focal spot size (μm)**	**Max pulse fluence (J/cm^2^)**	**Max peak intensity (W/cm^2^)**
8 ns	1064 nm	120	19.1	20,900	2.6 × 10^12^
8 ns	532 nm	32	9.5	22,500	2.8 × 10^12^
8 ns	355 nm	6	8.5	5,400	6.8 × 10^11^
8 ns	266 nm	6	7.6	7,200	9 × 10^11^
150 fs	795 nm	1	13.7	350	2.3 × 10^15^
150 fs	397 nm	0.012	8.2	11	7.6 × 10^13^
